# Effectiveness of music therapy in children with autism spectrum disorder: A systematic review and meta-analysis

**DOI:** 10.3389/fpsyt.2022.905113

**Published:** 2022-10-06

**Authors:** Xiaohua Ke, Wei Song, Minguang Yang, Jianhong Li, Weilin Liu

**Affiliations:** ^1^College of Rehabilitation Medicine, Fujian University of Traditional Chinese Medicine, Fuzhou, China; ^2^Department of Rehabilitation Medicine, Shanghai Fourth People’s Hospital Affiliated to Tongji University, Shanghai, China; ^3^Department of Rehabilitation Medicine, The First Affiliated Hospital of Fujian Medical University, Fuzhou, China

**Keywords:** music therapy, social interaction, speech, autism spectrum disorder, children

## Abstract

**Objectives:**

This study was to investigated the efficacy of music therapy (MT) in children with autism spectrum disorder (ASD) via a meta-analysis that comprehensively evaluated data from all eligible research in this field.

**Design:**

Systematic review and meta-analysis.

**Setting:**

A systematic search of the PubMed, Embase, and Cochrane Library databases from inception to October 2021 to identify studies that administered MT to children with ASD.

**Results:**

Eight randomized controlled trials (RCTs) including 608 participants met the inclusion criteria. The meta-analysis showed that MT was associated with a significant increase in social reactions among children with ASD (standardized mean difference (SMD) = 0.24, 95% confidence interval (CI) [0.03, 0.46], *I*^2^ = 0%, *P* = 0.03). However, MT did not elicit a significant increase in symptom severity (SMD = 0.17, 95% CI [−0.04,0.38], *I*^2^ = 0%,*P* = 0.12), social adaptive behavior (SMD = 0.02, 95% CI [−0.44,0.48], *I*^2^ = 0%,*P* = 0.93) or speech (SMD = 0.04, 95% CI [−0.39, 0.47], *I*^2^ = 0%, *P* = 0.86) in children with ASD.

**Conclusion:**

MT can improve social skills in children with ASD; however, there does not seem to be a consensus on the persistence of its effects. These findings can inform clinical practice. Promoting the use of MT in children with ASD and improving its symptoms are the ultimate goals.

## Introduction

Autism spectrum disorder (ASD) is a neurodevelopmental disorder of children, characterized by a behavioral phenotype of impaired social communication and stereotypic behavior (1). One percent of the worldwide population has ASD (1). Extensive genetic and early developmental environmental factors play an important role in the etiological heterogeneity of ASD. Functional neuroimaging is essential to highlight the presence of altered activation patterns in specific brain regions of patients with ASD, such as those involved in emotion regulation and social interaction (2), Frontal and cingulate cortices have been associated with persistently impaired social skills in ASD (3). Furthermore, inadequate local connectivity of the dorsal posterior cingulate cortex and the right medial paracentral lobule has been observed in ASD (4).

Music can trigger engagement in social functions, and musical activity is directly related to the fulfillment of basic human needs, such as communication, cooperation and social attachment (5). Supporting social functions was probably an important adaptive function of music in human evolution of humans (5). Despite their socioemotional impairment in everyday life, individuals with ASD have nearly normal abilities to recognize, experience and process emotional aspects of music (6). While listening to happy or sad music, individuals with ASD show activations in cortical and subcortical brain regions that are known to be deficient in this patient group with regard to nonmusical emotional stimuli (6).

As a cost effective, noninvasive adjunct to standard therapy, music therapy(MT) can be beneficial in the treatment of psychiatric disorders, in a variety of settings and patient groups, yet more validated scientific research is still required to establish MT as a quantified therapy (7). MT is usually easy to implement in practice (8). MT is a systematic intervention process in which the therapist helps the client to promote health, using musical experiences and the relationships that develop through them as dynamic forces of change (9). In terms of interventions, MT has been performed with child-centered or therapist-led approaches, using songs, improvisation, music listening, or combinations thereof (10). Some research has found that MT is an effective method with profound and consistent effects on improving the social skills of children with ASD (11).

Early social communication skills are theorized to be important for later more complex social behaviors (12). Social intervention from childhood onward is essential for individuals with autism. The neuropeptide oxytocin has been used as a potential therapy to reduce social impairment in ASD, but this hypothesis remains controversial and inconclusive (13). A placebo-controlled trial of intranasal oxytocin therapy in children and adolescents with ASD showed no significant between-group differences in the least-squares mean change from baseline on social functioning measures of over 24 weeks (14). Promising MT effects for autism have been shown in many domains (10), and MT may be a better option than some other treatments for improving the social skills of children with ASD.

This meta-analysis investigated the effects of MT on children with ASD. The primary outcome was the Social Reaction Scale (SRS) score, Gresham and Elliot created the SRS, which includes forms for parents, instructors, and students and is meant for three periods: preschool, elementary, and guidance school (11). The SRS mainly evaluates social functions; Phuong found that it exhibited good reliability with high internal consistency and test retest reliability, sensitivity, and specificity for identifying children with ASD (15). The secondary outcomes were the Vineland Adaptive Behavior Scales (VABS) and Verbal Production Evaluation Scale (VPES) socres. The VABS produces standardized scores in four domains: communication, social skills, daily living skills, and motor skills (16). Additionally, Hayoung designed the VPES to measure the participant production of target words according to the four speech components (17). The Autism Diagnostic Observation Schedule (ADOS) is a semistructured, standardized assessment of communication, social interaction, gaming, and restricted and repetitive behaviors (18). The Childhood Autism Rating Scale(CARS) is a useful tool for diagnosing children over 2 years old, and it has strong psychological measurement characteristics (19). The ADOS and CARS are measures of autism symptom severity, higher assessment result scores indicate greater symptom severity.

## Materials and methods

### Search strategy

The Preferred Reporting Items for System Reviews and Meta-Analyses (PRISMA) Statement Guide was reviewed. We searched the electronic databases that met the requirements and manually searched the reference list from the existing review. We searched PubMed, Embase, Cochrane Data, and Web of Science from database inception through October 4, 2021, using medical subject headings or a combination of free text words and concepts related to children with ASD and MT; the search was not limited by geography or publication type. Additional relevant publications were found by scanning the reference lists of the retrieved research.

### Inclusion criteria

The inclusion criteria were pre-specified according to the PICOS (participants, interventions, comparisons, outcomes, and study design) framework (see Table 1). Studies investigating children who had a definite diagnosis of autism were included. The interventions had to be delivered by a trained therapist to meet the definition of MT. Both active and receptive interventions were included. To attain a complete set of the variables tested in this context, no outcomes were indicated in the search syntax. Randomized controlled trials (RCTs) and controlled clinical trials were accepted.

**TABLE 1:** Eligibity criteria.

Domain	Inclusion criteria
**Domain**	**Inclusion criteria**
Patients	Children (below 14 years of age) were determined to have ASD
Interventions	Music therapy provided by a trained therapist
Comparators	Treatment as usual/Active control group
Outcomes	Not specified
Study designs	Randomized controlled trials/Controlled clinical trials

### Study selection and data extraction

All searched records were loaded into reference management software (Note Express V9.0) during the preliminary screening to minimize duplicates and discover probable acceptability by scanning titles and abstracts. The detailed strategy for each database is shown in the supplementary files as an online resource. After that, a full-text review was carried out. A reviewer was tasked with resolving all discrepancies. One reviewer used a prepared form to extract data, which was then confirmed by another reviewer. The details of the extracted data included: the first author name, study and participant characteristics, intervention(s) for the experimental and control groups, and outcomes.

### Assessment of the risk of bias in the included studies

The risk of bias in the included studies was evaluated by two independent reviewers (XK and WS) using the Cochrane Collaboration’s tool (20). This tool includes scores on six domains: (1) selective deviation (random sequence generation and allocation concealment), (2) performance deviation (participant and personnel blinding), (3) detection deviation (outcome assessor blinding), (4) attrition bias (incomplete outcome data), (5) reporting bias (selective reporting), and (6) other bias. Each item in each study was evaluated, and each domain was categorized as “low,” “high,” or “unclear” based on whether it matched the evaluation criteria for the feature conveyed by the items. Any discrepancies were resolved by a third reviewer.

## Data analysis

The statistical analyses were conducted using Review Manager 5.3 software (RevMan 5.3). When several scales were employed in each experiment, the combined statistics were the mean and standardized deviation (SD), standardized mean difference (SMD), and 95% confidence interval (CI). When the same scales were used in each trial, the combined statistics were the weighted mean difference and 95% CI. A random effect model was used to examine the data if the results had heterogeneity; otherwise, a fixed effect model was utilized. *I*^2^ reflects the study diversity. We contacted the author to request the original data if a study merely provided the value change in the evaluation scores. When two or more studies measured the same outcome and supplied data in a format suitable for pooling, the data were pooled for the meta-analysis. The χ^2^ test and Higgins’s I^2^ value were used to analyze the heterogeneity of the collected studies. *P* < 0.05 was considered significant when using the χ^2^ test. When data were available, the pooled effect was computed using the fixed-effect model, and no significant heterogeneity was found. In addition, the random-effect model was used.

## Results

### General results of the included studies

**FIGURE 1:**
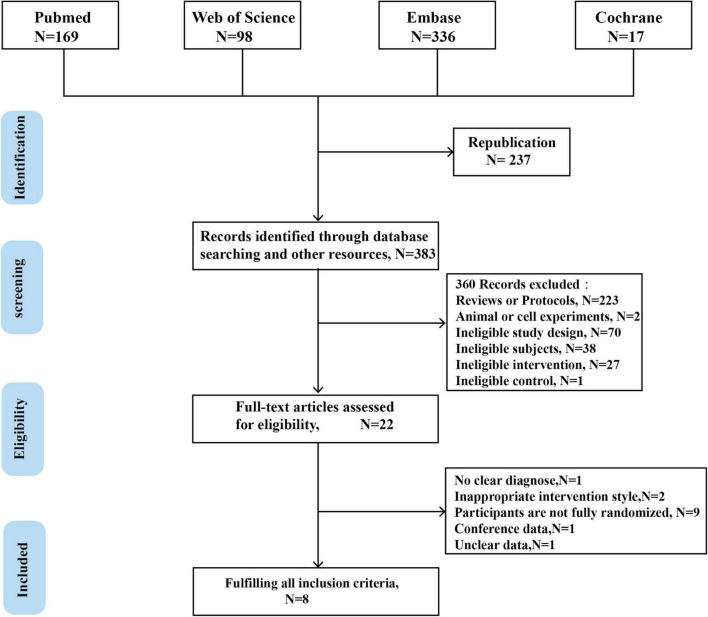
PRISMA flow chart. Showing the article selection process. Eight chosen articles.

From the four electronic databases, 608 records were found using the search method. After the duplicates were deleted, two reviewers evaluated the titles and abstracts and removed irrelevant entries. Finally, 22 full-text papers were reviewed for eligibility, with 8 studies meeting the requirements (11, 17, 21–26). Figure 1 shows the research selection flowchart for discovering eligible papers.

### Characteristics of the included studies

The major goal of this study was to investigate the overall effectiveness of MT for children with ASD across numerous outcomes and modifiers. In 8 trials, 608 persons met the inclusion criteria. Figure 1 shows the overall search strategy and article-screening procedure, whereas Table 2 shows the coded methodological, participant, and study features as well as the total treatment impact for each study.

**TABLE 2:** Study characteristics meeting inclusion criteria.

Study	*n*	Age	Duration	Setting	Control	measure	outcome
Rabeyron (23)	37	4–7 year	8 months, 30 min	MT	Music listening	CGI, CARS, ABC	Lethargy improved
Lim, (16)	50	3–5 year	3 days, 2 times/day	Music training	ST	VPES	No significance
Bieleninik (18)	364	4–6$\frac{11}{12}$ year	5 months	MT, Standard care	Standard care	ADOS,SRS	No significant difference
Gattino (19)	24	7–12 year	16 weeks	MT, Activities	Activities	CARS-BR	Improve nonverbal communication
Meghan (21)	51	6–12 year	8–12 weeks, 1times/week, 45 min	Music intervention	Non-music intervention	VABS,SRS-II	Improve social communication
Thompson (25)	33	3–6 year	16 weeks, 1 time/week, 30–40 min	MT, Early intervention	Early intervention	VSEEC, SRS-PS	Improve social interactions
Ghasemtabar (10)	27	7–12 year	2 months	MT	No MT	CARS, SRS	Enhance children’s social skills
Lim (20)	22	3–5 year	2 weeks, 3 days/week	MT, ABA(VB)	ST ABA (VB)	VPES	No significance

CGI, the Clinical Global Impression; CARS, Childhood Autism Rating Scale; ABC, the Aberrant Behavior Checklist; VPES, A verbal production evaluation scale; ADOS, Autism Diagnostic Observation Schedule; SRS, the Social Responsiveness Scale; VABS, Vineland Adaptive Behavior Scales; VSEEC, Vineland Social-Emotional Early Childhood Scales; ABA, Applied Behavior Analysis; ST, Speech Training.

The styles of music utilized in the intervention groups were diverse, involving commercial music (26), original music created by the therapist with speech training words (17), Orff-Schulwerk music (27), and other kinds of music styles. MT lasted anywhere from 3 days to 8 months in the studies that were included.

### Risk of bias in the included studies

**FIGURE 2:**
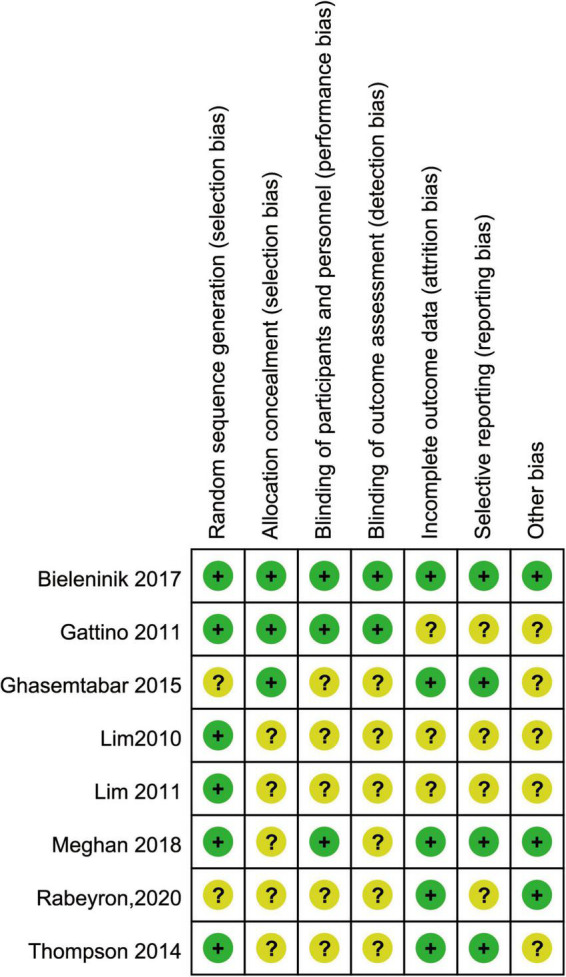
Risk of bias in the included studies.

The assessment of evidence quality was performed according to the Grading of Recommendations Assessment, Development and Evaluation (GRADE) process. Figure 2 provides a summary of the risk of bias results for each included study. Six trials (17, 21–24, 28) randomized their group assignments by using a computer-generated randomization list. One trial (11) matched the children by both age and sex to eliminate possible intervening variables. Three (11, 21, 22) of those studies reported allocation concealment. Three studies (10, 21, 22) described participant and personnel. Two studies (22, 24) clearly described the blind assessment of the outcome measures. The included studies were randomized controlled trials with high quality of evidence. Quality downgraded by one grade because of possible publication bias.

### Mean difference effect on symptoms severity in children with autism spectrum disorder

**FIGURE 3:**
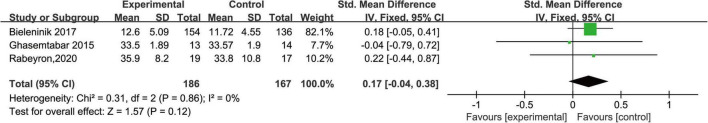
Forest plots of the effect of MT on symptoms severity compared with no MT; SD, standard deviation; 95% CI: 95% confidence interval; IV: inverse variance method.

In 8 studies, means and SD, or means and 95% CI were used to report the results before and after the interventions. The influence of MT on the overall level of functioning in children with ASD was assessed. The included literature used the ADOS and CARS to assess symptom severity in children with ASD. Two studies with 353 participants reported the relevant data. Compared with the control group, the level of intervention in the MT group did not significantly improve in either of the two studies. The pooled SMD did not show a statistically significant improving symptoms severity in children with ASD (*P* >0.05; Figure 3). MT did not improve symptom severity in children with ASD.

### Effect on the social reactions of children with autism spectrum disorder

**FIGURE 4:**
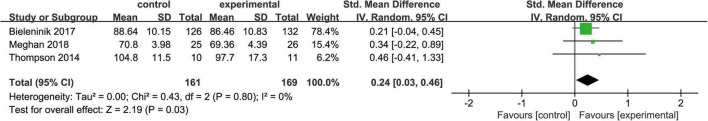
Forest plots of of MT (vs. no-MT) effect on the SRS score; SD: standard deviation; 95% CI: 95% confidence interval; IV: inverse variance method.

The MT effect on the social reactions of children with ASD was assessed in four studies. Three studies (21, 24, 28) with 330 participants reported the relevant data. Four studies used the SRS to measure the MT effect on children with ASD. One study (21) demonstrated a significantly lower post-intervention level of social interaction in the MT vs. the control group. The pooled mean difference (MD) showed a statistically significant increase in the SRS score (*P* < 0.05; Figure 4). MT improved the social reactions of children with ASD.

### Effect on the adaptive social behaviors of children with autism spectrum disorder

**FIGURE 5:**
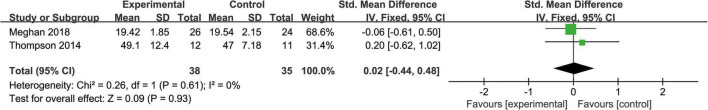
Forest plots of the MT (vs. no-MT) effect on social adaptation. SD: standard deviation; 95% CI: 95% confidence interval; IV: inverse variance method.

Two studies with 34 participants reported the relevant data. Both used the VABS to measure the MT effect on children with ASD. The difference was significant between music and other training types (*P* <0.05). The pooled MD did not show a statistically significant increase in adaptive social behaviors. (*P* >0.05; Figure 5). MT did not improve social adaptive behavior.

### Effect on the speech of children with autism spectrum disorder

**FIGURE 6:**
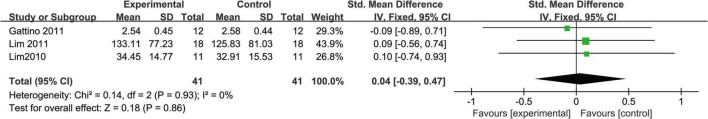
Forest plots of the MT (vs. no-MT) effect on speech. SD: standard deviation; 95% CI: 95% confidence interval; IV: inverse variance method.

Three studies (17, 22, 23) with 82 participants reported relevant data. Three studies used the VPES or the CARS–BR (Brazilian–Portuguese version) scale to measure the MT effect on children with ASD. The CARS–BR aspects assessed in this study included verbal, nonverbal and social communication (22). We analyzed the results of the verbal subscale of the CARS-BR, which showed that the difference was not significant between music and other training types. The pooled SMD did not show a statistically significant increase in speech (*P* >0.05; Figure 6). MT did not improve the speech of children with ASD.

## Discussion

Unlike previous meta-analysis, our subjects were children younger than 12 years old. Some limitations continued through to the meta-analysis and should be considered. First, the study design, methodological quality, and types and forms of music contributed to increasing the heterogeneity of the meta-analyses. The goal of this meta-analysis was to determine how MT affected children with ASD. This meta-analysis included eight RCTs with 608 individuals who compared MT groups to no-MT control groups. MT had a positive impact on social skills in children with ASD.

Social skills were examined in three studies. The subjects were of different ages, including preschoolers and school-aged children. Two of them explicitly used improvisational MT, and both required parental involvement. One was solo therapy by a professional therapist, without specifying musical form. The intervention frequency was weekly, with no significant differences in duration. The Intervention periods were different, ranging from 8 weeks to 5 months. Because differences in the age at intervention and the presence or absence of parents can have an effect on children’s MT outcomes; although I^2^ was 0%, we believe this result is likely to be heterogeneous. With a growing body of research pointing to the potential benefits of parent-mediated interventions for supporting development in children with ASD, there is both a need, and increasing capacity, to examine influencing factors (29).

Karin found the MT relationship to be an important predictor of the development of social skills (30). Regarding the effect on the social reactions in children with ASD, the MT group showed a significant difference from the no MT group. Music is known to regulate arousal and attention in the brain and can engage different areas in the brains of individuals with neurological conditions (31). MT may increase the socioemotional motivation of children with ASD. Deficits in socioemotional reciprocity; nonverbal communicative actions utilized for social engagement; and the formation, maintenance, and comprehension of relationships are all examples of social communication impairments in children with ASD (32). Children with such impairments are unlikely to engage in social interactions. In addition, their social problems will not gradually improve as the children develop. Because their environments become more complicated, the children increase their understanding of their social discomfort, and may experience increased damage and pain during adolescence. The MT group did not show a significant difference from the no-MT group. A social adaptive function is required for children to adapt to their environments and interact adequately with others. Defects in social skills may result in limited relationships with peers and family members. Early social and communication skills are essential for a better developmental trajectory and the acquisition of more complicated abilities later in life (33).

People with ASD usually have a unique attraction to music and may have enhanced musical ability. This attraction to music can be used to allow children with ASD to participate in musical experiences, thereby promoting social skills (34). The musical strengths of children on the autism spectrum not only compensate for their social difficulties but also provide the potential for lifelong engagement in and enjoyment of musical activity (35). Many children with ASD respond favorably to music, finding it a safe and controlled stimulus for social interaction and social skill development. Musical stimuli may aid in the development of social interactions for a variety of reasons. Music has been shown to engage brain networks that are involved in similar musical and nonmusical tasks, and to maximize target behaviors through synchronized neuronal firings. MT interventions have a positive impact on social skills, including increasing participation (36), improving social connection and increasing emotional involvement (25). MT has also been shown to improve social greeting rituals and joint attention (37) as well as communication skills, peer interactions, and cognitive social skills (27). These studies show that MT can lead to measurable improvements in the social relationships of children with ASD.

Mean difference did not significantly decrease symptoms in children with autism. The ADOS is the medical standard for diagnosing autism and has been effective for classifying autism, but it is less specific and sometimes less sensitive for distinguishing children with mild autism (38). The CARS is widely used to detect and diagnose autism, and it has shown a strong concordance with the DSM–IV criteria for autism (39). However, the CARS is subjective. Moreover, the treatment durations included in the studies were relatively short, with none lasting more than 1 year. More research time and objective assessment methods may be needed to determine whether MT can improve symptom severity in children with autism. Although *I*^2^ was 0%, we believe this result was likely to be heterogeneous. Social impairment is the core symptom of children with autism. The improvement of social ability should improve its severity, which requires larger samples and more studies to prove. Adaptive behavior was measured using reports from the parent/caregiver and teacher forms on the VABS, A total of two articles were analyzed. The children in the two studies were of different ages. One with parental involvement and one without although *I*^2^ was 0%, we believe this is likely to be heterogeneous. Parents not only play an important role in the early diagnosis of the child, but also occupy an irreplaceable position in the later intervention treatment. Parental involvement contributes to the development of parent-child relationships and the development of children’s early social interaction skills.

Speech function in children with autism was assessed in two papers by the same authors using scales created by the authors themselves. The two studies had different ages of children and different interventions, and one used behavior analytic therapy. Although *I*^2^ was 0%, we believe this is likely to be heterogeneous. Social communication difficulties are a key feature of ASD and must be present to receive a behavioral diagnosis. Speech is one aspect of social communication that encompasses a wide range of abilities. Children with ASD are more likely to show developmental impairments in speech and communication (40). They struggle with maintaining a conversation, extending welcomes and farewells, taking turns appropriately, and applying conversational repair procedures. These shortcomings are linked to difficulties with perspective-taking and the theory of mind. Because pragmatic speech problems are very common in ASD, this area has received much attention. When compared to peers with usual development, intellectual disabilities, or other disorders, children with ASD have reduced pragmatic skills.

Mean difference is thought to be an excellent way to help children with ASD improve their speech and communication abilities (17). Music instruction was found to be useful in improving the speech production of 50 children with ASD, including semantics, phonology, pragmatics, and prosody (17). Therefore, music, especially music suitable for a child’s age and developmental level, has been used as a consistent and reliable strategy to improve speech and language and to cultivate communication skills in almost all treatment methods. However, the results of another study indicated (23) that both music and speech training are effective for language for ASD children, and the differences between music and language training were not statistically significant.

Music is an auditory stimulus that interests and motivates many children with ASD (41). MT improves movement synchrony in children with ASD (42). Children with high levels of adaptive behavior or low levels of maladaptive behavior displayed greater exercise intensity during a fast music condition (43). MT for motor function training in children with ASD has been less studied, but the impact of MT is broad, and more difficult to quantify. Targeted assessment tools are required to evaluate the impact and long-term effects of MT.

A second music-based assessment for children with ASD is the Individual Music-Centered Assessment Profile for Neurodevelopmental Disorders (IMCAP-ND) (44). This assessment is part of the development framework based on the relationship between MT and ASD. In this framework, the ability to perform, and interpret creative music is evaluated. This assessment is based on music-centric treatment, which provides information about how people play a role in music, which helps with non-music interaction and understanding.

## Limitation

Failure to pre-register the protocol for this review is a problem because it introduces a potential bias to the evaluation. Fewer articles were included in this paper and only those published in English were covered, which may be subject to publication bias. Relatively little research has been conducted on the effects of MT on children with ASD and much of what has been published lacks scientific rigor. Fewer articles have been published in recent years, so selective publication can’t be ruled out.

## Conclusion

In summary, our findings suggest that MT is effective in improving the social interaction of children with ASD. This convenient, short-term music program may help children with ASD learn social skills and integrate into society. Because of the small number of eligible studies, the conclusions should be applied with caution, and there appears to be no consensus on the continuation of the intervention effects. More assessor-blinded, international, parallel-group, pragmatic RCTs are needed to prove the effectiveness of MT in improving social interaction.

As an effective early intervention, MT, through auditory action on the cerebral system and other brain regions, adjusts the cerebral cortex, enhances emotions and arousal levels, and has a unique treatment effect on autistic children’s cognition, emotions and behaviors. To make MT truly beneficial for the majority of children with ASD, several changes should be implemented—including reductions in the required physical and mental effort, increases in the number of randomized controlled trials on the topic, and further exploration of the mechanism underlying MT—to elucidate its psychological mechanism. These suggestions can enhance the use of MT with children with ASD.

## Data availability statement

The original contributions presented in this study are included in the article/supplementary material, further inquiries can be directed to the corresponding author.

## Author contributions

WL contributed to conceiving and designing the study. XK and WS drafted the manuscript. MY and JL conducted the literature search and extracted the data. All authors carried out with the direct participation of the study.
